# Supplementary material for: A Systematic Review and Meta-Analysis: Lactobacillus acidophilus for Treating Acute Gastroenteritis in Children
Source: Nutrients. 2022 Feb 6;14(3):682. doi: 10.3390/nu14030682 (PMC8839062; doi:10.3390/nu14030682)
Supplement: Supplementary file 1 [file nutrients-14-00682-s001.zip › nutrients-1536305-supplementary.pdf]

SUPPORTING INFORMATION

Table S1. Search strategy.
Table S2. Characteristics of the included studies.
Table S3. Characteristics of the excluded trials.
Table S4. GRADE analysis for selected outcomes.

Figure S1. Risk of bias in the included studies.
Figure S2. Funnel plot of comparison: *Lactobacillus acidophilus* vs. control. Duration of diarrhoea.

Figure S3. *Lactobacillus acidophilus* vs. control. Duration of diarrhoea. Bacterial species (individual probiotic strains *Lactobacillus acidophilus* vs *Lactobacillus acidophilus* + *Bifidobacterium* vs *Lactobacillus acidophilus* + *Lactobacillus rhamnosus vs Lactobacillus acidophilus* + other probiotic strains).
Figure S4. *Lactobacillus acidophilus* vs. control. Duration of diarrhoea. Dose of *Lactobacillus acidophilus* in individual probiotic strains studies (high dose [$\geq 10^{10}$ CFU/day] vs lower dose [$<10^{10}$CFU/day]).
Figure S5. *Lactobacillus acidophilus* vs. control. Duration of diarrhoea. Setting (studies carried out in geographical Asia vs Europe vs America).
Figure S6. *Lactobacillus acidophilus* vs. control. Duration of diarrhoea. Aetiology of diarrhoea (Rotavirus-positive 100% vs Rotavirus-positive 1%~99% vs Unknown aetiology).

Figure S7. *Lactobacillus acidophilus* vs. control. Methodology. Randomisation.
Figure S8. *Lactobacillus acidophilus* vs. control. Methodology. Allocation concealment.
Figure S9. *Lactobacillus acidophilus* vs. control. Methodology. Blinding
Figure S10. *Lactobacillus acidophilus* vs. control. Methodology. Incomplete outcome data
Figure S11. *Lactobacillus acidophilus* vs. control. Frequency of stools

Table S1. Search strategy

Pubmed

(((probiotics[Title/Abstract] OR probiotic[Title/Abstract] OR Lactobacillus acidophilus[Title/Abstract] OR Lactobacillus amylovorus[Title/Abstract] OR Acidophilus[Title/Abstract]) AND (diarrhea[Title/Abstract] OR diarrhoe*[Title/Abstract] OR diarhe*[Title/Abstract] OR dysenter*[Title/Abstract] OR gastro enteritis[Title/Abstract] OR diarrhea[Title/Abstract] OR diarrh*[Title/Abstract] OR gastritis[Title/Abstract] OR gastrit*[Title/Abstract] OR gastroenteritis[Title/Abstract] OR gastroenterocolitis[Title/Abstract] OR vomit*[Title/Abstract] OR intestinal infection*[Title/Abstract] OR gastrointestinal infection*[Title/Abstract] OR rotavirus[Title/Abstract])) AND (children[Title/Abstract] OR child*[Title/Abstract] OR infants[Title/Abstract] OR infant*[Title/Abstract] OR toddler*[Title/Abstract] OR adolescent*[Title/Abstract] OR teenage*[Title/Abstract] OR baby[Title/Abstract] OR preschool children[Title/Abstract])) AND (randomized controlled trial[pt] OR controlled clinical trial[pt] OR randomized[tiab] OR placebo[tiab] OR drug therapy[sh] OR randomly[tiab] OR trial[tiab] OR groups[tiab] NOT (animals [mh] NOT humans [mh]))

EMBASE

#1. 'lactobacillus acidophilus'/exp

#2. (('probiotics'/exp OR 'probiotic'/exp OR 'lactobacillus'/exp) AND acidophilus OR 'lactobacillus'/exp) AND amylovorus OR acidophilus:ab,ti

#3. #1 OR #2

#4. 'acute gastroenteritis'/exp

#5. ((('diarrhea'/exp OR diarrhoe* OR diarhe* OR dysenter* OR 'gastro'/exp) AND 'enteritis'/exp OR 'diarrhea'/exp OR diarrh* OR 'gastritis'/exp OR gastrit* OR 'gastroenteritis'/exp OR 'gastroenterocolitis'/exp OR vomit* OR intestinal) AND infection* OR gastrointestinal) AND infection* OR rotavirus:ab,ti

#6. #4 OR #5

#7. 'child'/exp

#8. ('children'/exp OR children OR child* OR 'infants'/exp OR infants OR infant* OR toddler* OR adolescent* OR teenage* OR 'baby'/exp OR baby OR 'preschool'/exp OR preschool) AND children:ab,ti

#9. #7 OR #8

#10. 'random':ab,ti OR 'placebo':ab,ti OR 'double-blind':ab,ti

#11. #3 AND #6 AND #9 AND #10

Cochrane Library

#1 MeSH descriptor: [Lactobacillus acidophilus] explode all trees

#2 (probiotics OR probiotic OR Lactobacillus acidophilus OR Lactobacillus amylovorus OR Acidophilus):ti,ab,kw (Word variations have been searched)

#3 #1 OR #2 7536

#4 MeSH descriptor: [] explode all trees

#5 (diarrhea OR diarrhoe* OR diarhe* OR dysenter* OR gastro enteritis OR diarrhea OR diarrh* OR gastritis OR gastrit* OR gastroenteritis OR gastroenterocolitis OR vomit* OR intestinal infection* OR gastrointestinal infection* OR rotavirus):ti,ab,kw (Word variations have been searched)

#6 MeSH descriptor: [Child] explode all trees

#7 (children OR child* OR infants OR infant* OR toddler* OR adolescent* OR teenage* OR baby OR preschool children):ti,ab,kw (Word variations have been searched)

#8 #6 OR #7 283367

#9 #3 AND #5 AND #8

Table S2. Characteristics of the included studies

Ref..	Study ID (Country)	FU	Exp/ Cont	Patients	Intervention	Daily dose (CFU)	Comparison	Inclusion criteria/ definition of diarrhoea	Outcomes (probiotic vs. Placebo)	Funding
**14**	Abolfazl 2018 (Iran)	91.05% (173/190)	86/87	2 y – 10 y	Bifidobacterium infantis, Lactobacillus casei, Lactobacillus acidophilus, Lactobacillus rhamnosus, Lactobacillus bulgaricus, B. infantis, Bifidobacterium brevis, and Streptococcus thermophilus	?	placebo	noninflammatory acute diarrhea	Duration of diarrhea (days) (mean ± SD)<br>11.1 ± 32.3 VS 7.1 ± 43.4; p = .001<br>Frequency of diarrhea (mean ± SD)<br>Day 2: 23.2 ± 48.7 VS 45.2 ± 9.9; p < .001<br>Day 4: 46.1 ± 8.2 VS 48.2 ± 71.4; p < .001	Not stated
**6**	Badriul 2015 (Indonesia)	100.00%	56/56	6 mo – 36 mo	ORS + zinc + Lactobacillus (L.) rhamnosus R0011 and L. acidophilus R0052, for 7 days	$1×10^8$	ORS + zinc	(semi-) watery stools (Bristol criteria > type 4) lasting ≥48 h	Median diarrhea duration (hours)(range)<br>68.5 (13 – 165) (n = 56) VS 61.5 (21 – 166) (n = 56); p = 0.596	Dexa Medica and Indofarma
**11**	Eduardo 2007 (Peru)	96.25% (77/80)	40/37	3 mo - 4 y	Viatol + Lacteol + killed Lactobacillus LB strain and neutralized supernatant spent culture medium	$3x10^{10}$	salicylic acid, banana and orange flavor, sucrose, and yellow and brown iron oxides	with a duration of <72 hours and with 3 or more watery stools within the previous 24 hours	Duration of diarrhea (hours) (median (quartile 1/quartile 3))<br>16.6 (7.1 - 50.3) (n = 40) VS 10.0 (6.0 - 56.7) (n = 37); p = 0.275	Not stated

**16**	Ener 2013 (Turke)	92.48% (209/226)	113/96	3 mo - 10 y	ORS and/or intravenous therapy + live bacteria including Lactobacillus acidophilus, Lactobacillus rhamnosus, Bifidobacterium bifidum, Bifidobacterium longum, Enterococcus faecium, for 5 days	< 2.5x 10^9	ORS and/or intravenous therapy	acute diarrhea (>4 (semi)watery stools/day according to Bristol criteria (Bristol criteria ≥6)) at least lasting more than 12 h and less than 72 h	Duration of diarrhea (hours) (mean ± SD)<br>77.9 ± 30.5 VS 114.6 ± 37.4; p < 0.0001<br>Frequency of stools per day<br>24th hour: 3.93 ± 2.2 VS 6.43 ± 3.4<br>48th hour: 3.38 ± 1.6 VS 4.65 ± 3.61<br>72th hour: 2.95 ± 1.2 VS 3.18 ± 2.4<br>96th hour: 2.18 ± 1.6 VS 2.64 ± 2.3<br>120th hour: 1.91 ± 1.0 VS 1.76 ± 1.61	Not stated
**17**	K. Chen 2020 (China)	89.81% (194/216)	96/98	1 y - 3 y	ORS + zinc + B. lactis Bi-07, L. rhamnosus HN001, and L. acidophilus NCFM), once a day	?	ORS + zinc	at least one episode of mild to moderate acute diarrhoea [>4 (semi)watery stools per day] lasting more than 12 h and less than 72 h	Duration of diarrhea (hours) (mean ± SD)<br>121.4 ± 13.7 (n=96) VS 143.9 ± 19.8 (n=98); p < 0.001<br>Diarrhoea frequency (mean ± SD)<br>24th hour: 5.1 ± 1.8 VS 5.3 ± 1.7; p = 0.427<br>48th hour: 4.4 ± 1.3 VS 4.7 ± 1.9; p = 0.202<br>72th hour: 2.7 ± 0.5 VS 3.6 ± 0.7; p < 0.001	Not stated
**18**	MING 2001 (TaiWan)	100.00%	50/50	6 mo - 60 mo	Lactobacillus acidophilus, Bifidobacterium infantis	1x10^9	placebo	duration of diarrhea less than 5 days + watery stools more	Duration of diarrhea in hospital (days) (mean ± SD)	Not stated

								than 3 times during 24 hours	3.1 ± 0.7 VS 3.6 ± 0.8; p < .001<br>Diarrhea frequency (mean ± SD)<br>Day 1: 2.9 ± 2.0 VS 4.0 ± 1.3; p < .001<br>Day 2: 1.9 ± 1.9 VS 3.7 ± 2.4; p < .001	
**7**	Myeong 2017 (Korea)	100.00%	28/29	9 mo - 16 mo	Bifidobacterium longum BORI and Lactobacillus acidophilus AD031, twice a day, for 3 days	$1x10^9$	placebo	diagnosed as infected with rotavirus via a latex agglutination test	Duration of diarrhea (days) (mean ± SD)<br>4.38 ± 1.29 VS 5.61 ± 1.23; p = 0.001	Not stated
**19**	Raanan 2005 (Israel)	100.00%	33/32	6 mo - 12 mo	ORS + zine + Streptococcus thermophilus, Bifidobacterium lactis, Lactobacillus acidophilus	$6x10^9$	ORS + zine + fructo-oligosaccharides	four or more watery stools in 24 hours for more than one day and less than 7 days	Time to resolution of diarrhea (days) (mean ± SD)<br>1.34 ± 0.71 VS 1.97 ± 1.24; p = 0.017	part by Remedia, Israel
**20**	Rafeey 2008 (Iran)	100.00%	40/40	12.3 mo - 26.8 mo	L.acidophilus， 2 capsules per day	$1x10^{10}$	placebo	3 or more watery stool per day for less than 48 h	Hospital stay (days) (mean ± SD)<br>3.4 ± 0.9 VS 4.0 ± 1.1<br>Stool frequency (mean ± SD)<br>day 1: 3.7 ± 3 VS 3.8 ± 3.3<br>day 2: 4.0 ± 3.2 VS 4.0 ± 3.6<br>day 3: 1.4 ± 2.6 VS 2.3 ± 2.6	The Liver an Gastrointestinal Research Center of the Tabriz Medical University

**21**	Sandra 2020 (Belgium)	76.67% (46/60)	26/20	3 mo - 186 mo	ORS + Streptococcus thermophilus; Lactobacillus rhamnosus + Lactobacillus acidophilus; Bifidobacterium infantis + Bifidobacterium lactis, for 7 days	< 6.5x 10^9	ORS	mild to moderate acute diarrhea (>4 [semi] watery stools⁄day) according to the Bristol criteria (Bristol criteria ≥6), since at least 1 day and lasting within 7 days	Duration of diarrhea (days) (mean ± SD)<br>3.04 ± 1.36 VS 4.20 ± 1.34; p = 0.018	Not stated
**22**	Simakachorn 2000 (Thailand)	100.00%	37/36	3 mo - 24 mo	ORS + lyophilized heat-killed L. acidophilus LB at 12-hour intervals for five doses, 48-hour treatment	$2x10^{10}$	placebo	acute watery diarrhea present for no more than 5 days and mild or moderate dehydration	Mean duration of diarrhea (hours) (mean ± SD)<br>no antibiotic: 42.9 ± 27.2 VS 74.0 ± 42.9; p = 0.016	Merck Ltd., Bangkok, Thailand
**5**	Tran 2018 (Vietnam)	95.71% (290/303)	143/147	11.8 mo - 21.5 mo	L. acidophilus twice daily, for 5 days	$4x10^8$	placebo	loose or watery stools at least three times in a 24-hour period that did not contain blood or mucus with a history of less than three days	The first 24-hour diarrhea free period (hours) ((median (IQR))<br>35 (20 - 68) (n = 143) VS 43 (15 - 66) (n = 147); p = 1.62	The Wellcome Trust and the OAK Foundation
**12**	Vandenplas 2011 (Belgium)	100.00%	57/54	3 mo - 186 mo	ORS + Composition of Probiotical (Streptococcus thermophilus, Lactobacillus rhamnosus +	< 6.5x 10^9	ORS	three or more liquid or loose stools per day lasting for less than or equal	Duration of diarrhoea (days) (median(range))<br>3 (1 - 6) (n = 57) VS 4 (2 - 6) (n = 54); p < 0.005<br>Number of stools ⁄ day (Median) (IQ25-75)	Phacobel Belgium

					Lactobacillus acidophilus, Bifidobacterium infantis + Bifidobacterium lactis), for 7 days			to 7 days	Day 1: 5 (4 - 6) VS 5 (4 - 6); p = N.S.<br>Day 2: 3 (2 - 4) VS 4 (2 - 5); p = 0.04<br>Day 3: 2 (1 - 3) VS 2 (2 - 3); p = N.S.<br>Day 4: 2 (1-3) VS 2 (1-3); p = N.S.	
**23**	Vanessa 2007 (France)	100%	42/38	1 mo - 24 mo	1 sachet every 12 hours (heat-killed L acidophilus LB per sachet), 72-hour treatment	$2x10^{10}$	ORS	at least 4 liquid stools passed during the last 24 hours	Duration of diarrhea (hours) (mean ± SD)<br>39.5 ± 10.5 VS 63.4 ± 14.9	Not stated
**24**	Vikrant 2005 （India）	90.82% (89/98)	48/50	6 mo - 12 y	ORS + LA， for 3 days	$1.5x 10^{10}$	ORS	admitted to diarrhea training and treatment unit (DTTU) with acute watery diarrhea	Duration diarrhea (hours) (mean ± SD)<br>51.97 ± 1.44 VS 53.97 ± 1.48; p = 0.96<br>No. of stools (mean ± SD)<br>24 hours: 16.33 ± 2.02 VS 14.91 ± 2.09; p = 1.09<br>24~48 hours: 6.47 ± 2.66 VS 4.68 ± 3.46; p = 1.38	Not stated

*Reference numbers as in the main manuscript

[#] Strain designation confirmed by the manufacturer and/or the authors.

FU, follow up; ORS, oral rehydration solution

Table S3. Characteristics of the excluded trials

Ref.*	Author, year of publication	Reason for exclusion
1	Boulloche 1994 (France)	Not in English
2	Han 2004 (Korea)	Not in English
3	Río 2004 (not mentioned)	Not in English
4	Rerksuppaphol 2010 (Thailand)	Missing data, just reported IQR
5	Giuseppe 2010 (Bolivia)	Missing data, just reported IQR
6	Jamie 2015 (USA)	Miss the primary outcome
7	Marion 2013 (Vietnam)	Study Protocol
8	Veereman 2009 (not mentioned)	Crohn's disease

* In the supplementary materials

Table S4. GRADE analysis for selected outcomes.

Lactobacillus acidophilus compared to placebo/no treatment for acute gastroenteritis in children

Patient or population: acute gastroenteritis in children
Setting:
Intervention: Lactobacillus acidophilus
Comparison: placebo/no treatment

Outcomes	**Anticipated absolute effects*** (95% CI)		Relative effect (95% CI)	№ of participants (studies)	Certainty of the evidence (GRADE)	Comments
	**Risk with placebo/no treatment**	**Risk with Lactobacillus acidophilus**				
Duration of diarrhoea	The mean duration of diarrhoea ranged from **1.75 to 7.71** days	MD **0.69 days lower** (1.04 lower to 0.33 lower)	-	1765 (15 RCTs)	⊕⊕⊕◯ MODERATE [a,b]	
Frequency of diarrhoea - Day 2	The mean duration of diarrhoea ranged from 3.67 **to 7.1 days**	MD **0.45 days lower** (1.22 lower to 0.33 lower)	-	965 (7 RCTs)	⊕⊕◯◯ LOW [c, d]	
Frequency of diarrhoea - Day 3	The mean duration of diarrhoea ranged from 2.3 **to 3.6 days**	MD **0.61 days lower** (1.00 lower to 0.23 lower)	-	594 (4 RCTs)	⊕⊕◯◯ LOW [c, d]	

Frequency of diarrhoea - Day 5	The mean duration of diarrhoea was **1.76 days**	MD **0.15 days higher** (0.22 lower to 0.52 higher)	-	209 (1 RCTs)	⨁⨁◯◯ LOW [c]

***The risk in the intervention group** (and its 95% confidence interval) is based on the assumed risk in the comparison group and the **relative effect** of the intervention (and its 95% CI).

CI: Confidence interval; **MD:** Mean difference.

GRADE Working Group grades of evidence
High certainty: We are very confident that the true effect lies close to that of the estimate of the effect
Moderate certainty: We are moderately confident in the effect estimate: The true effect is likely to be clos

e to the estimate of the effect, but there is a possibility that it is substantially different
Low certainty: Our confidence in the effect estimate is limited: The true effect may be substantially different from the estimate of the effect
Very low certainty: We have very little confidence in the effect estimate: The true effect is likely to be substantially different from the estimate of effect

Explanations

a. Substantial heterogeneity $I^2 = 94\%$ ($p < 0.00001$), not explained by the subgroup analyses

b. A different definition of diarrhoea across the studies

c. In one study, no blinding ensured, and the sample size is relatively large

d. Large heterogeneity (>50%; $p < 0.05$)

a.

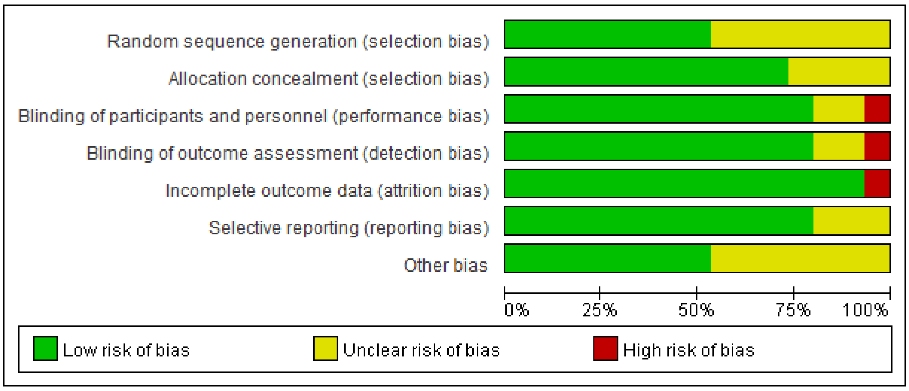

b.

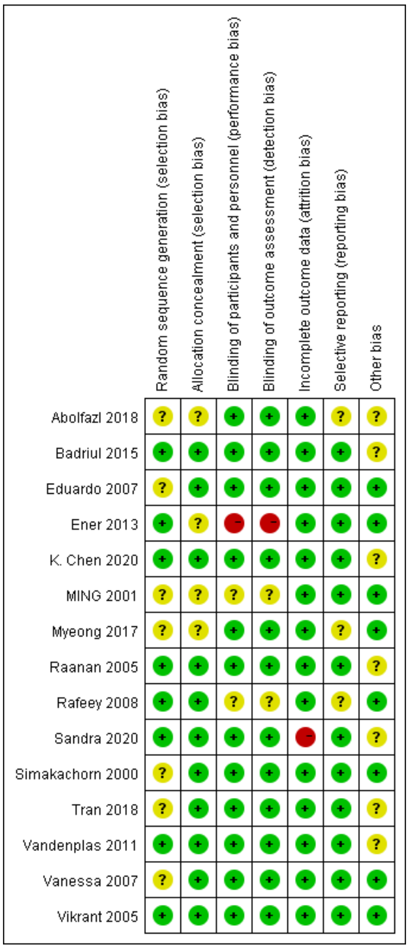

Figure S1. Risk of bias in the included studies. (**a**) Risk of bias graph: review authors' judgements about each risk of bias item presented as percentages across all included studies; (**b**) Risk of bias summary: review authors' judgements about each risk of bias item for each included study.

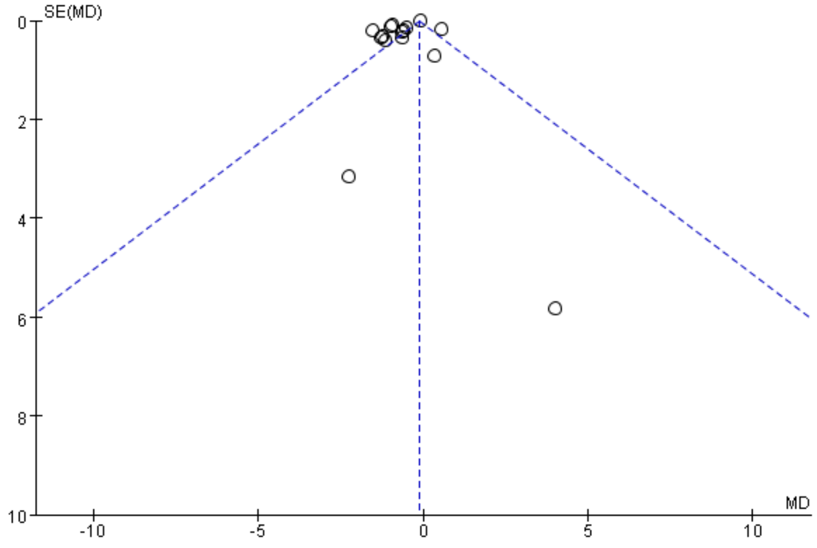


```
Begg's Test

  adj. Kendall's Score (P-Q) =        29
          Std. Dev. of Score =     20.21
           Number of Studies =        15
                          z  =      1.44
                    Pr > |z| =     0.151
                          z  =      1.39 (continuity corrected)
                    Pr > |z| =     0.166 (continuity corrected)
```

Egger's test

Std_Eff	Coef.	Std. Err.	t	P>|t|	[95% Conf. Interval]	
slope	-.059639	.0442868	-1.35	0.201	-.1553148	.0360368
bias	-2.695705	.9659362	-2.79	0.015	-4.782484	-.6089272

Figure S2. Funnel plot of comparison: *Lactobacillus acidophilus* vs. control. Duration of diarrhoea

Study or Subgroup	Experimental Mean	Experimental SD	Experimental Total	Control Mean	Control SD	Control Total	Weight	Mean Difference IV, Random, 95% CI
**2.6.1 Lactobacillus acidophilus**								
Eduardo 2007	2.49	1.38	40	3.14	1.63	37	6.9%	-0.65 [-1.33, 0.03]
Rafeey 2008	3.4	0.9	40	4	1.1	40	8.1%	-0.60 [-1.04, -0.16]
Simakachorn 2000	1.79	1.13	37	3.08	1.79	36	6.9%	-1.29 [-1.98, -0.60]
Tran 2018	2.31	1.5	143	1.75	1.59	147	8.5%	0.56 [0.20, 0.92]
Vanessa 2007	1.65	0.44	42	2.64	0.62	38	8.9%	-0.99 [-1.23, -0.75]
Vikrant 2005	2.17	0.06	48	2.25	0.06	50	9.3%	-0.08 [-0.10, -0.06]
**Subtotal (95% CI)**			**350**			**348**	**48.6%**	**-0.47 [-0.95, 0.01]**
Heterogeneity: Tau² = 0.31; Chi² = 87.95, df = 5 (P < 0.00001); I² = 94%								
Test for overall effect: Z = 1.91 (P = 0.06)								
**2.6.2 Lactobacillus acidophilus+Bifidobacterium**								
MING 2001	3.1	0.7	50	3.6	0.8	50	8.7%	-0.50 [-0.79, -0.21]
Myeong 2017	4.38	1.29	28	5.61	1.23	29	7.0%	-1.23 [-1.88, -0.58]
**Subtotal (95% CI)**			**78**			**79**	**15.8%**	**-0.80 [-1.51, -0.10]**
Heterogeneity: Tau² = 0.20; Chi² = 3.97, df = 1 (P = 0.05); I² = 75%								
Test for overall effect: Z = 2.23 (P = 0.03)								
**2.6.3 Lactobacillus acidophilus+Lactobacillus rhamnosus**								
Badriul 2015	5.45	8.27	56	7.71	22.24	56	0.3%	-2.26 [-8.47, 3.95]
**Subtotal (95% CI)**			**56**			**56**	**0.3%**	**-2.26 [-8.47, 3.95]**
Heterogeneity: Not applicable								
Test for overall effect: Z = 0.71 (P = 0.48)								
**2.6.4 Lactobacillus acidophilus + other probiotic strains**								
Abolfazl 2018	11.1	32.3	86	7.1	43.4	87	0.1%	4.00 [-7.39, 15.39]
Ener 2013	3.25	1.27	113	4.78	1.56	96	8.3%	-1.53 [-1.92, -1.14]
K. Chen 2020	5.06	0.57	96	6	0.83	98	9.0%	-0.94 [-1.14, -0.74]
Raanan 2005	1.34	0.71	33	1.97	1.24	32	7.9%	-0.63 [-1.12, -0.14]
Sandra 2020	3.04	1.36	26	4.2	1.34	20	6.3%	-1.16 [-1.95, -0.37]
Vandenplas 2011	4.35	4.59	57	4	2.97	54	3.7%	0.35 [-1.08, 1.78]
**Subtotal (95% CI)**			**411**			**387**	**35.3%**	**-0.97 [-1.36, -0.57]**
Heterogeneity: Tau² = 0.12; Chi² = 13.92, df = 5 (P = 0.02); I² = 64%								
Test for overall effect: Z = 4.81 (P < 0.00001)								
**Total (95% CI)**			**895**			**870**	**100.0%**	**-0.69 [-1.04, -0.33]**
Heterogeneity: Tau² = 0.35; Chi² = 238.27, df = 14 (P < 0.00001); I² = 94%								
Test for overall effect: Z = 3.83 (P = 0.0001)								
Test for subgroup differences: Chi² = 2.67, df = 3 (P = 0.44), I² = 0%								

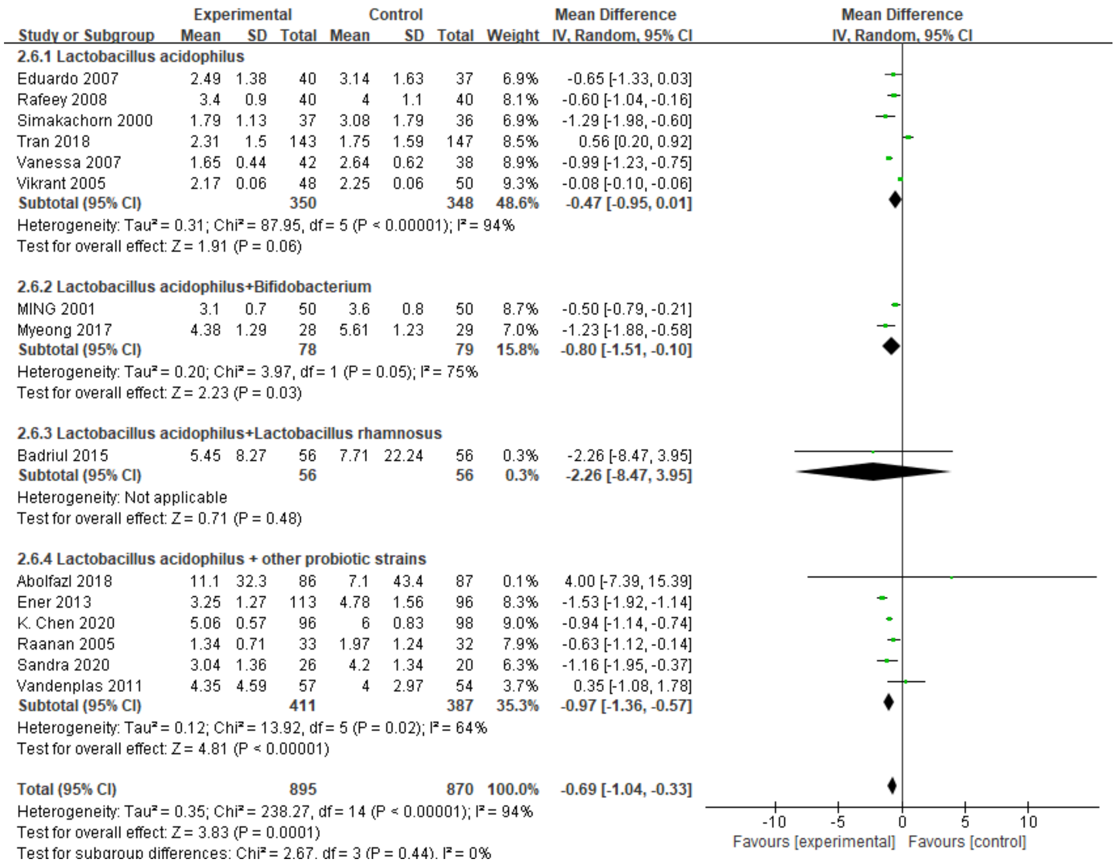

Figure S3. *Lactobacillus acidophilus* vs. control. Duration of diarrhoea. Bacterial species (individual probiotic strains *Lactobacillus acidophilus* vs *Lactobacillus acidophilus* + *Bifidobacterium* vs *Lactobacillus acidophilus* + *Lactobacillus rhamnosus vs Lactobacillus acidophilus* + other probiotic strains)

Study or Subgroup	Experimental Mean	Experimental SD	Experimental Total	Control Mean	Control SD	Control Total	Weight	Mean Difference IV, Random, 95% CI
**2.3.1 individual strains - Lactobacillus acidophilus ⩾ 10^9CFU**								
Eduardo 2007	2.49	1.38	40	3.14	1.63	37	14.0%	-0.65 [-1.33, 0.03]
Rafeey 2008	3.4	0.9	40	4	1.1	40	16.7%	-0.60 [-1.04, -0.16]
Simakachorn 2000	1.79	1.13	37	3.08	1.79	36	13.9%	-1.29 [-1.98, -0.60]
Vanessa 2007	1.65	0.44	42	2.64	0.62	38	18.5%	-0.99 [-1.23, -0.75]
Vikrant 2005	2.17	0.06	48	2.25	0.06	50	19.4%	-0.08 [-0.10, -0.06]
**Subtotal (95% CI)**			**207**			**201**	**82.5%**	**-0.69 [-1.25, -0.14]**
Heterogeneity: Tau² = 0.34; Chi² = 75.08, df = 4 (P < 0.00001); I² = 95%								
Test for overall effect: Z = 2.45 (P = 0.01)								
**2.3.2 individual strains - Lactobacillus acidophilus < 10^9CFU**								
Tran 2018	2.31	1.5	143	1.75	1.59	147	17.5%	0.56 [0.20, 0.92]
**Subtotal (95% CI)**			**143**			**147**	**17.5%**	**0.56 [0.20, 0.92]**
Heterogeneity: Not applicable								
Test for overall effect: Z = 3.09 (P = 0.002)								
**Total (95% CI)**			**350**			**348**	**100.0%**	**-0.47 [-0.95, 0.01]**
Heterogeneity: Tau² = 0.31; Chi² = 87.95, df = 5 (P < 0.00001); I² = 94%								
Test for overall effect: Z = 1.91 (P = 0.06)								
Test for subgroup differences: Chi² = 13.89, df = 1 (P = 0.0002), I² = 92.8%								

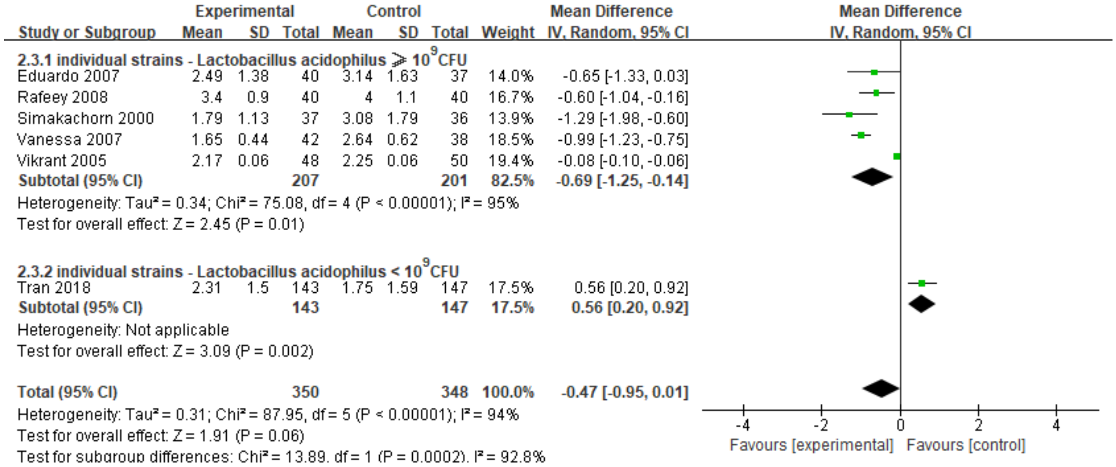

Figure S4. *Lactobacillus acidophilus* vs. control. Duration of diarrhoea. Dose of *Lactobacillus acidophilus* in individual probiotic strains studies (high dose [≥ 10^9 CFU/day] vs lower dose [<10^9CFU/day])

Study or Subgroup	Experimental Mean	Experimental SD	Experimental Total	Control Mean	Control SD	Control Total	Weight	Mean Difference IV, Random, 95% CI
**2.4.1 Asia**								
Abolfazl 2018	11.1	32.3	86	7.1	43.4	87	0.1%	4.00 [-7.39, 15.39]
Badriul 2015	5.45	8.27	56	7.71	22.24	56	0.3%	-2.26 [-8.47, 3.95]
Ener 2013	3.25	1.27	113	4.78	1.56	96	8.3%	-1.53 [-1.92, -1.14]
K. Chen 2020	5.06	0.57	96	6	0.83	98	9.0%	-0.94 [-1.14, -0.74]
MING 2001	3.1	0.7	50	3.6	0.8	50	8.7%	-0.50 [-0.79, -0.21]
Myeong 2017	4.38	1.29	28	5.61	1.23	29	7.0%	-1.23 [-1.88, -0.58]
Raanan 2005	1.34	0.71	33	1.97	1.24	32	7.9%	-0.63 [-1.12, -0.14]
Rafeey 2008	3.4	0.9	40	4	1.1	40	8.1%	-0.60 [-1.04, -0.16]
Simakachorn 2000	1.79	1.13	37	3.08	1.79	36	6.9%	-1.29 [-1.98, -0.60]
Tran 2018	2.31	1.5	143	1.75	1.59	147	8.5%	0.56 [0.20, 0.92]
Vikrant 2005	2.17	0.06	48	2.25	0.06	50	9.3%	-0.08 [-0.10, -0.06]
**Subtotal (95% CI)**			**730**			**721**	**74.2%**	**-0.66 [-1.07, -0.26]**
Heterogeneity: Tau² = 0.34; Chi² = 175.61, df = 10 (P < 0.00001); I² = 94%								
Test for overall effect: Z = 3.21 (P = 0.001)								
**2.4.2 Europe**								
Sandra 2020	3.04	1.36	26	4.2	1.34	20	6.3%	-1.16 [-1.95, -0.37]
Vandenplas 2011	4.35	4.59	57	4	2.97	54	3.7%	0.35 [-1.08, 1.78]
Vanessa 2007	1.65	0.44	42	2.64	0.62	38	8.9%	-0.99 [-1.23, -0.75]
**Subtotal (95% CI)**			**125**			**112**	**18.9%**	**-0.88 [-1.41, -0.36]**
Heterogeneity: Tau² = 0.10; Chi² = 3.52, df = 2 (P = 0.17); I² = 43%								
Test for overall effect: Z = 3.29 (P = 0.0010)								
**2.4.3 America**								
Eduardo 2007	2.49	1.38	40	3.14	1.63	37	6.9%	-0.65 [-1.33, 0.03]
**Subtotal (95% CI)**			**40**			**37**	**6.9%**	**-0.65 [-1.33, 0.03]**
Heterogeneity: Not applicable								
Test for overall effect: Z = 1.88 (P = 0.06)								
**Total (95% CI)**			**895**			**870**	**100.0%**	**-0.69 [-1.04, -0.33]**
Heterogeneity: Tau² = 0.35; Chi² = 238.27, df = 14 (P < 0.00001); I² = 94%								
Test for overall effect: Z = 3.83 (P = 0.0001)								
Test for subgroup differences: Chi² = 0.49, df = 2 (P = 0.78), I² = 0%								

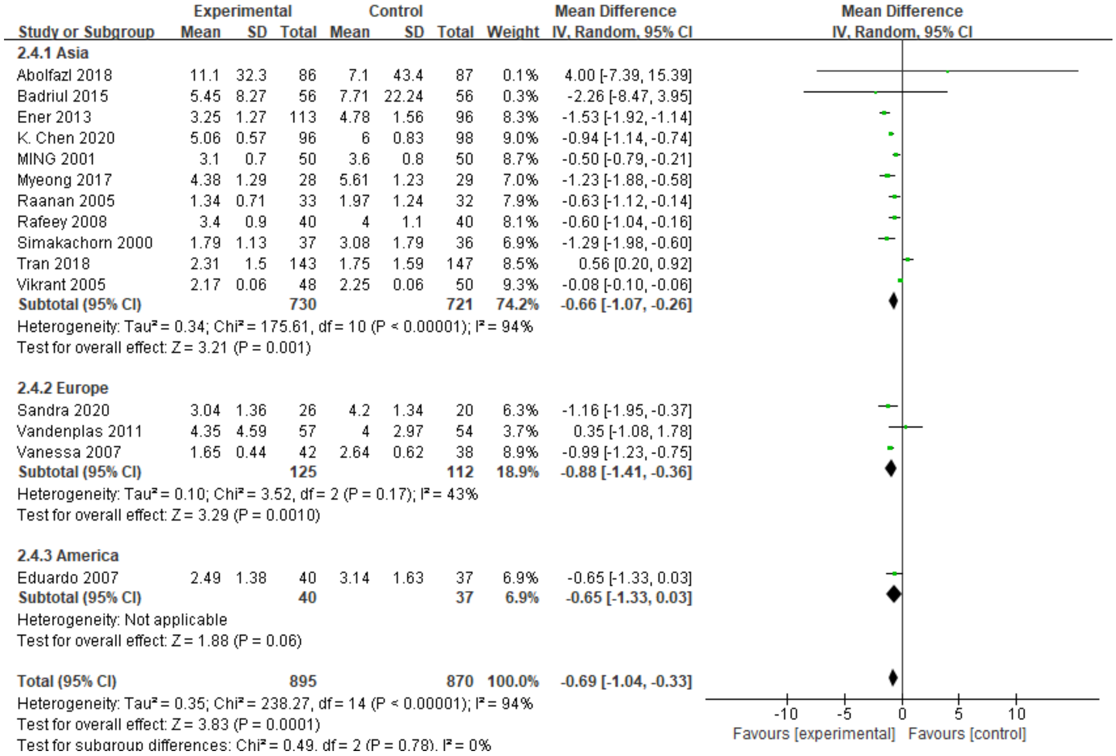

Figure S5. *Lactobacillus acidophilus* vs. control. Duration of diarrhoea. Setting (studies carried out in geographical Asia vs Europe vs America

Study or Subgroup	Experimental Mean	Experimental SD	Experimental Total	Control Mean	Control SD	Control Total	Weight	Mean Difference IV, Random, 95% CI
**2.5.1 Rotavirus-positive 100%**								
Myeong 2017	4.38	1.29	28	5.61	1.23	29	7.1%	-1.23 [-1.88, -0.58]
**Subtotal (95% CI)**			**28**			**29**	**7.1%**	**-1.23 [-1.88, -0.58]**
Heterogeneity: Not applicable								
Test for overall effect: Z = 3.68 (P = 0.0002)								
**2.5.2 Rotavirus-positive 1%~99%**								
Eduardo 2007	2.49	1.38	40	3.14	1.63	37	7.0%	-0.65 [-1.33, 0.03]
MING 2001	3.1	0.7	50	3.6	0.8	50	9.0%	-0.50 [-0.79, -0.21]
Simakachorn 2000	1.79	1.13	37	3.08	1.79	36	6.9%	-1.29 [-1.98, -0.60]
Tran 2018	2.31	1.5	143	1.75	1.59	147	8.7%	0.56 [0.20, 0.92]
Vanessa 2007	1.65	0.44	42	2.64	0.62	38	9.2%	-0.99 [-1.23, -0.75]
Vikrant 2005	51.97	1.44	48	53.97	1.48	50	7.5%	-2.00 [-2.58, -1.42]
**Subtotal (95% CI)**			**360**			**358**	**48.4%**	**-0.79 [-1.43, -0.15]**
Heterogeneity: Tau² = 0.58; Chi² = 77.57, df = 5 (P < 0.00001); I² = 94%								
Test for overall effect: Z = 2.40 (P = 0.02)								
**2.5.3 Unknown aetiology**								
Abolfazl 2018	11.1	32.3	86	7.1	43.4	87	0.1%	4.00 [-7.39, 15.39]
Badriul 2015	5.45	8.27	56	7.71	22.24	56	0.3%	-2.26 [-8.47, 3.95]
Ener 2013	3.25	1.27	113	4.78	1.56	96	8.6%	-1.53 [-1.92, -1.14]
K. Chen 2020	5.06	0.57	96	6	0.83	98	9.4%	-0.94 [-1.14, -0.74]
Raanan 2005	1.34	0.71	33	1.97	1.24	32	8.0%	-0.63 [-1.12, -0.14]
Rafeey 2008	3.4	0.9	40	4	1.1	40	8.3%	-0.60 [-1.04, -0.16]
Sandra 2020	3.04	1.36	26	4.2	1.34	20	6.4%	-1.16 [-1.95, -0.37]
Vandenplas 2011	4.35	4.59	57	4	2.97	54	3.5%	0.35 [-1.08, 1.78]
**Subtotal (95% CI)**			**507**			**483**	**44.5%**	**-0.91 [-1.24, -0.58]**
Heterogeneity: Tau² = 0.10; Chi² = 16.88, df = 7 (P = 0.02); I² = 59%								
Test for overall effect: Z = 5.38 (P < 0.00001)								
**Total (95% CI)**			**895**			**870**	**100.0%**	**-0.84 [-1.18, -0.51]**
Heterogeneity: Tau² = 0.31; Chi² = 103.65, df = 14 (P < 0.00001); I² = 86%								
Test for overall effect: Z = 4.89 (P < 0.00001)								
Test for subgroup differences: Chi² = 1.01, df = 2 (P = 0.60), I² = 0%								

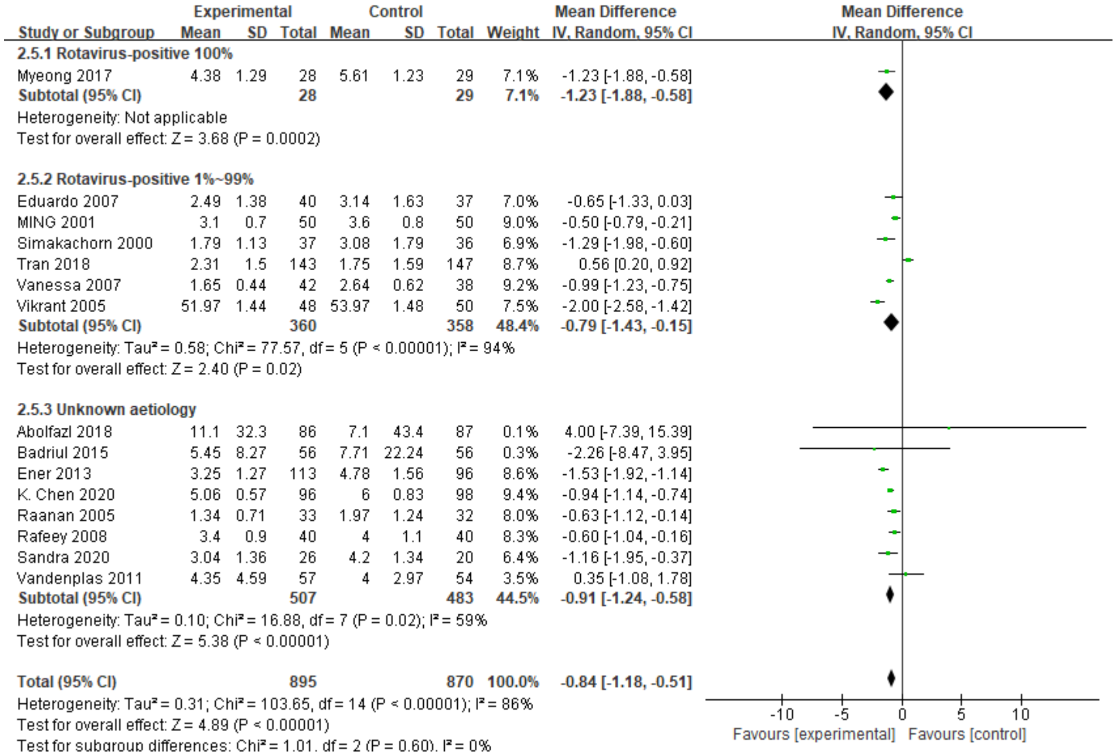

Figure S6. *Lactobacillus acidophilus* vs. control. Duration of diarrhoea. Aetiology of diarrhoea (Rotavirus-positive 100% vs Rotavirus-positive 1%~99% vs Unknown aetiology)

Study or Subgroup	Experimental Mean	Experimental SD	Experimental Total	Control Mean	Control SD	Control Total	Weight	Mean Difference IV, Random, 95% CI
**3.1.1 Adequate**								
Badriul 2015	5.45	8.27	56	7.71	22.24	56	0.3%	-2.26 [-8.47, 3.95]
Ener 2013	3.25	1.27	113	4.78	1.56	96	8.3%	-1.53 [-1.92, -1.14]
K. Chen 2020	5.06	0.57	96	6	0.83	98	9.0%	-0.94 [-1.14, -0.74]
Raanan 2005	1.34	0.71	33	1.97	1.24	32	7.9%	-0.63 [-1.12, -0.14]
Rafeey 2008	3.4	0.9	40	4	1.1	40	8.1%	-0.60 [-1.04, -0.16]
Sandra 2020	3.04	1.36	26	4.2	1.34	20	6.3%	-1.16 [-1.95, -0.37]
Vandenplas 2011	4.35	4.59	57	4	2.97	54	3.7%	0.35 [-1.08, 1.78]
Vikrant 2005	2.17	0.06	48	2.25	0.06	50	9.3%	-0.08 [-0.10, -0.06]
**Subtotal (95% CI)**			**469**			**446**	**53.0%**	**-0.73 [-1.25, -0.21]**
Heterogeneity: Tau² = 0.41; Chi² = 139.29, df = 7 (P < 0.00001); I² = 95%								
Test for overall effect: Z = 2.75 (P = 0.006)								
**3.1.2 Inadequate/unclear**								
Abolfazl 2018	11.1	32.3	86	7.1	43.4	87	0.1%	4.00 [-7.39, 15.39]
Eduardo 2007	2.49	1.38	40	3.14	1.63	37	6.9%	-0.65 [-1.33, 0.03]
MING 2001	3.1	0.7	50	3.6	0.8	50	8.7%	-0.50 [-0.79, -0.21]
Myeong 2017	4.38	1.29	28	5.61	1.23	29	7.0%	-1.23 [-1.88, -0.58]
Simakachorn 2000	1.79	1.13	37	3.08	1.79	36	6.9%	-1.29 [-1.98, -0.60]
Tran 2018	2.31	1.5	143	1.75	1.59	147	8.5%	0.56 [0.20, 0.92]
Vanessa 2007	1.65	0.44	42	2.64	0.62	38	8.9%	-0.99 [-1.23, -0.75]
**Subtotal (95% CI)**			**426**			**424**	**47.0%**	**-0.64 [-1.21, -0.07]**
Heterogeneity: Tau² = 0.44; Chi² = 59.67, df = 6 (P < 0.00001); I² = 90%								
Test for overall effect: Z = 2.22 (P = 0.03)								
**Total (95% CI)**			**895**			**870**	**100.0%**	**-0.69 [-1.04, -0.33]**
Heterogeneity: Tau² = 0.35; Chi² = 238.27, df = 14 (P < 0.00001); I² = 94%								
Test for overall effect: Z = 3.83 (P = 0.0001)								
Test for subgroup differences: Chi² = 0.05, df = 1 (P = 0.83), I² = 0%								

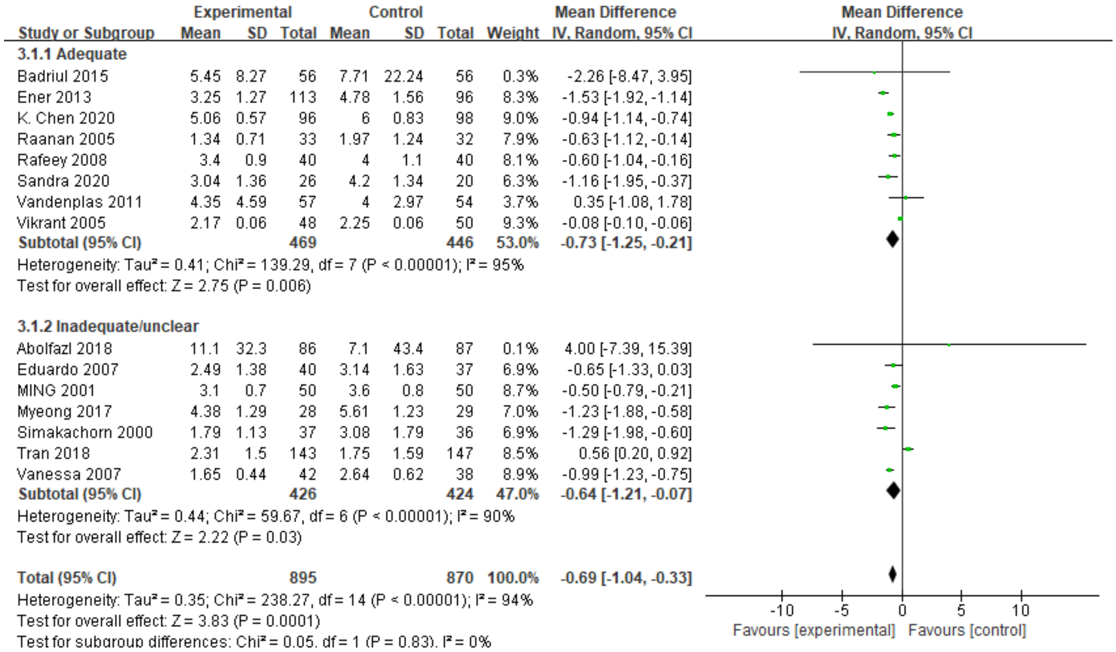

Figure S7. *Lactobacillus acidophilus* vs. control. Methodology. Randomisation

Study or Subgroup	Experimental Mean	Experimental SD	Experimental Total	Control Mean	Control SD	Control Total	Weight	Mean Difference IV, Random, 95% CI
**3.2.1 Adequate**								
Badriul 2015	5.45	8.27	56	7.71	22.24	56	0.3%	-2.26 [-8.47, 3.95]
Eduardo 2007	2.49	1.38	40	3.14	1.63	37	6.9%	-0.65 [-1.33, 0.03]
K. Chen 2020	5.06	0.57	96	6	0.83	98	9.0%	-0.94 [-1.14, -0.74]
Raanan 2005	1.34	0.71	33	1.97	1.24	32	7.9%	-0.63 [-1.12, -0.14]
Rafeey 2008	3.4	0.9	40	4	1.1	40	8.1%	-0.60 [-1.04, -0.16]
Sandra 2020	3.04	1.36	26	4.2	1.34	20	6.3%	-1.16 [-1.95, -0.37]
Simakachorn 2000	1.79	1.13	37	3.08	1.79	36	6.9%	-1.29 [-1.98, -0.60]
Tran 2018	2.31	1.5	143	1.75	1.59	147	8.5%	0.56 [0.20, 0.92]
Vandenplas 2011	4.35	4.59	57	4	2.97	54	3.7%	0.35 [-1.08, 1.78]
Vanessa 2007	1.65	0.44	42	2.64	0.62	38	8.9%	-0.99 [-1.23, -0.75]
Vikrant 2005	2.17	0.06	48	2.25	0.06	50	9.3%	-0.08 [-0.10, -0.06]
**Subtotal (95% CI)**			**618**			**608**	**75.8%**	**-0.57 [-0.96, -0.18]**
Heterogeneity: Tau² = 0.32; Chi² = 168.63, df = 10 (P < 0.00001); I² = 94%								
Test for overall effect: Z = 2.86 (P = 0.004)								
**3.2.2 Inadequate/unclear**								
Abolfazl 2018	11.1	32.3	86	7.1	43.4	87	0.1%	4.00 [-7.39, 15.39]
Ener 2013	3.25	1.27	113	4.78	1.56	96	8.3%	-1.53 [-1.92, -1.14]
MING 2001	3.1	0.7	50	3.6	0.8	50	8.7%	-0.50 [-0.79, -0.21]
Myeong 2017	4.38	1.29	28	5.61	1.23	29	7.0%	-1.23 [-1.88, -0.58]
**Subtotal (95% CI)**			**277**			**262**	**24.2%**	**-1.05 [-1.77, -0.33]**
Heterogeneity: Tau² = 0.36; Chi² = 18.77, df = 3 (P = 0.0003); I² = 84%								
Test for overall effect: Z = 2.85 (P = 0.004)								
**Total (95% CI)**			**895**			**870**	**100.0%**	**-0.69 [-1.04, -0.33]**
Heterogeneity: Tau² = 0.35; Chi² = 238.27, df = 14 (P < 0.00001); I² = 94%								
Test for overall effect: Z = 3.83 (P = 0.0001)								
Test for subgroup differences: Chi² = 1.31, df = 1 (P = 0.25), I² = 23.7%								

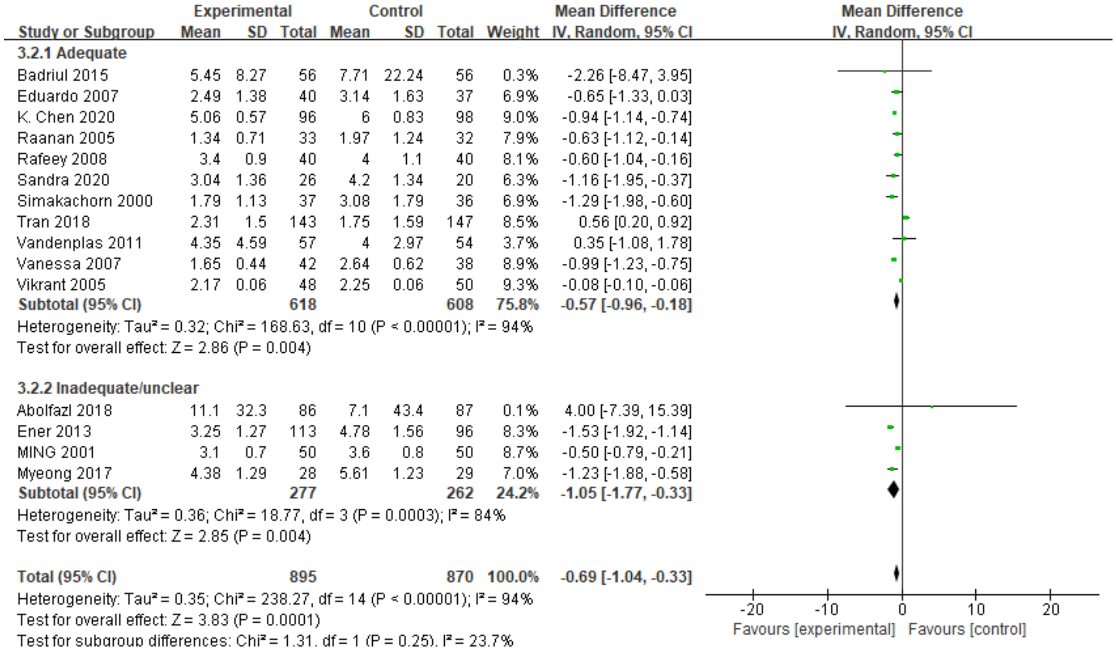

Figure S8. *Lactobacillus acidophilus* vs. control. Methodology. Allocation concealment

Study or Subgroup	Experimental Mean	Experimental SD	Experimental Total	Control Mean	Control SD	Control Total	Weight	Mean Difference IV, Random, 95% CI
**3.3.1 Adequate**								
Abolfazl 2018	11.1	32.3	86	7.1	43.4	87	0.1%	4.00 [-7.39, 15.39]
Badriul 2015	5.45	8.27	56	7.71	22.24	56	0.3%	-2.26 [-8.47, 3.95]
Eduardo 2007	2.49	1.38	40	3.14	1.63	37	6.9%	-0.65 [-1.33, 0.03]
K. Chen 2020	5.06	0.57	96	6	0.83	98	9.0%	-0.94 [-1.14, -0.74]
Myeong 2017	4.38	1.29	28	5.61	1.23	29	7.0%	-1.23 [-1.88, -0.58]
Raanan 2005	1.34	0.71	33	1.97	1.24	32	7.9%	-0.63 [-1.12, -0.14]
Sandra 2020	3.04	1.36	26	4.2	1.34	20	6.3%	-1.16 [-1.95, -0.37]
Simakachorn 2000	1.79	1.13	37	3.08	1.79	36	6.9%	-1.29 [-1.98, -0.60]
Tran 2018	2.31	1.5	143	1.75	1.59	147	8.5%	0.56 [0.20, 0.92]
Vandenplas 2011	4.35	4.59	57	4	2.97	54	3.7%	0.35 [-1.08, 1.78]
Vanessa 2007	1.65	0.44	42	2.64	0.62	38	8.9%	-0.99 [-1.23, -0.75]
Vikrant 2005	2.17	0.06	48	2.25	0.06	50	9.3%	-0.08 [-0.10, -0.06]
**Subtotal (95% CI)**			**692**			**684**	**74.8%**	**-0.62 [-1.03, -0.22]**
Heterogeneity: Tau² = 0.35; Chi² = 175.62, df = 11 (P < 0.00001); I² = 94%								
Test for overall effect: Z = 3.01 (P = 0.003)								
**3.3.3 Inadequate/unclear**								
Ener 2013	3.25	1.27	113	4.78	1.56	96	8.3%	-1.53 [-1.92, -1.14]
MING 2001	3.1	0.7	50	3.6	0.8	50	8.7%	-0.50 [-0.79, -0.21]
Rafeey 2008	3.4	0.9	40	4	1.1	40	8.1%	-0.60 [-1.04, -0.16]
**Subtotal (95% CI)**			**203**			**186**	**25.2%**	**-0.87 [-1.52, -0.23]**
Heterogeneity: Tau² = 0.29; Chi² = 18.21, df = 2 (P = 0.0001); I² = 89%								
Test for overall effect: Z = 2.64 (P = 0.008)								
**Total (95% CI)**			**895**			**870**	**100.0%**	**-0.69 [-1.04, -0.33]**
Heterogeneity: Tau² = 0.35; Chi² = 238.27, df = 14 (P < 0.00001); I² = 94%								
Test for overall effect: Z = 3.83 (P = 0.0001)								
Test for subgroup differences: Chi² = 0.41, df = 1 (P = 0.52), I² = 0%								

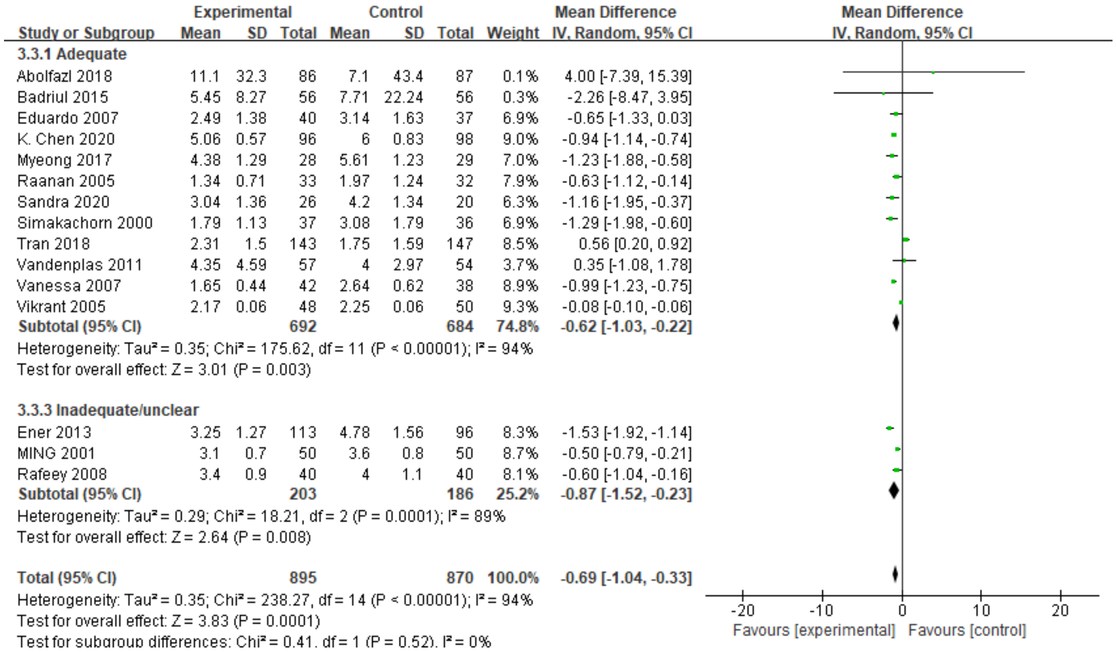

Figure S9. *Lactobacillus acidophilus* vs. control. Methodology. Blinding

Study or Subgroup	Experimental Mean	Experimental SD	Experimental Total	Control Mean	Control SD	Control Total	Weight	Mean Difference IV, Random, 95% CI
**3.4.1 Adequate**								
Badriul 2015	5.45	8.27	56	7.71	22.24	56	0.3%	-2.26 [-8.47, 3.95]
MING 2001	3.1	0.7	50	3.6	0.8	50	8.7%	-0.50 [-0.79, -0.21]
Myeong 2017	4.38	1.29	28	5.61	1.23	29	7.0%	-1.23 [-1.88, -0.58]
Raanan 2005	1.34	0.71	33	1.97	1.24	32	7.9%	-0.63 [-1.12, -0.14]
Rafeey 2008	3.4	0.9	40	4	1.1	40	8.1%	-0.60 [-1.04, -0.16]
Simakachorn 2000	1.79	1.13	37	3.08	1.79	36	6.9%	-1.29 [-1.98, -0.60]
Vandenplas 2011	4.35	4.59	57	4	2.97	54	3.7%	0.35 [-1.08, 1.78]
Vanessa 2007	1.65	0.44	42	2.64	0.62	38	8.9%	-0.99 [-1.23, -0.75]
**Subtotal (95% CI)**			**343**			**335**	**51.5%**	**-0.78 [-1.04, -0.53]**
Heterogeneity: Tau² = 0.06; Chi² = 13.97, df = 7 (P = 0.05); I² = 50%								
Test for overall effect: Z = 5.94 (P < 0.00001)								
**3.4.2 Inadequate/unclear**								
Abolfazl 2018	11.1	32.3	86	7.1	43.4	87	0.1%	4.00 [-7.39, 15.39]
Eduardo 2007	2.49	1.38	40	3.14	1.63	37	6.9%	-0.65 [-1.33, 0.03]
Ener 2013	3.25	1.27	113	4.78	1.56	96	8.3%	-1.53 [-1.92, -1.14]
K. Chen 2020	5.06	0.57	96	6	0.83	98	9.0%	-0.94 [-1.14, -0.74]
Sandra 2020	3.04	1.36	26	4.2	1.34	20	6.3%	-1.16 [-1.95, -0.37]
Tran 2018	2.31	1.5	143	1.75	1.59	147	8.5%	0.56 [0.20, 0.92]
Vikrant 2005	2.17	0.06	48	2.25	0.06	50	9.3%	-0.08 [-0.10, -0.06]
**Subtotal (95% CI)**			**552**			**535**	**48.5%**	**-0.60 [-1.15, -0.04]**
Heterogeneity: Tau² = 0.43; Chi² = 145.21, df = 6 (P < 0.00001); I² = 96%								
Test for overall effect: Z = 2.11 (P = 0.04)								
**Total (95% CI)**			**895**			**870**	**100.0%**	**-0.69 [-1.04, -0.33]**
Heterogeneity: Tau² = 0.35; Chi² = 238.27, df = 14 (P < 0.00001); I² = 94%								
Test for overall effect: Z = 3.83 (P = 0.0001)								
Test for subgroup differences: Chi² = 0.37, df = 1 (P = 0.55), I² = 0%								

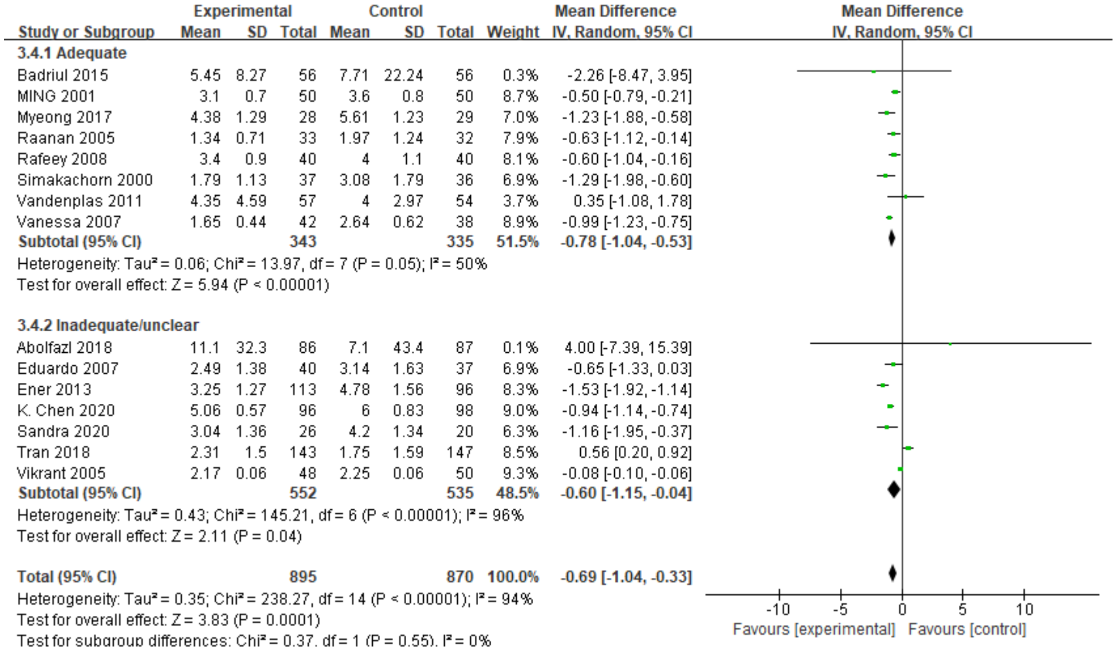

Figure S10. *Lactobacillus acidophilus* vs. control. Methodology. Incomplete outcome data

Study or Subgroup	Experimental Mean	Experimental SD	Experimental Total	Control Mean	Control SD	Control Total	Weight	Mean Difference IV, Random, 95% CI
**4.1.1 Diarrhoea on day 1**								
Ener 2013	3.93	2.2	113	6.43	3.4	96	5.4%	-2.50 [-3.29, -1.71]
K. Chen 2020	5.1	1.8	96	5.3	1.7	0		Not estimable
MING 2001	2.9	2	50	4	1.3	50	5.9%	-1.10 [-1.76, -0.44]
Rafeey 2008	3.7	3	40	3.8	3.3	40	3.4%	-0.10 [-1.48, 1.28]
Vandenplas 2011	5	1.48	57	5	1.48	54	6.3%	0.00 [-0.55, 0.55]
Vikrant 2005	16.33	2.02	48	14.91	2.09	50	5.3%	1.42 [0.61, 2.23]
**Subtotal (95% CI)**			**404**			**290**	**26.2%**	**-0.47 [-1.71, 0.78]**
Heterogeneity: Tau² = 1.83; Chi² = 52.52, df = 4 (P < 0.00001); I² = 92%								
Test for overall effect: Z = 0.73 (P = 0.46)								
**4.1.2 Diarrhoea on day 2**								
Abolfazl 2018	11.1	32.3	86	7.1	43.4	87	0.1%	4.00 [-7.39, 15.39]
Ener 2013	3.38	1.6	113	4.65	3.61	96	5.4%	-1.27 [-2.05, -0.49]
K. Chen 2020	4.4	1.3	96	4.7	1.9	98	6.6%	-0.30 [-0.76, 0.16]
MING 2001	1.9	1.9	50	3.7	2.4	50	5.1%	-1.80 [-2.65, -0.95]
Rafeey 2008	4	3.2	40	4	3.6	40	3.1%	0.00 [-1.49, 1.49]
Vandenplas 2011	3	1.48	57	3.67	2.22	54	5.7%	-0.67 [-1.38, 0.04]
Vikrant 2005	6.47	2.66	48	4.68	3.46	50	3.9%	1.79 [0.57, 3.01]
**Subtotal (95% CI)**			**490**			**475**	**29.9%**	**-0.45 [-1.22, 0.33]**
Heterogeneity: Tau² = 0.72; Chi² = 28.15, df = 6 (P < 0.0001); I² = 79%								
Test for overall effect: Z = 1.13 (P = 0.26)								
**4.1.3 Diarrhoea on day 3**								
Ener 2013	2.95	1.2	113	3.18	2.4	96	6.3%	-0.23 [-0.76, 0.30]
K. Chen 2020	2.7	0.5	96	3.6	0.7	98	7.3%	-0.90 [-1.07, -0.73]
Rafeey 2008	1.4	2.6	40	2.3	2.6	40	4.1%	-0.90 [-2.04, 0.24]
Vandenplas 2011	2	1.48	57	2.43	0.86	54	6.6%	-0.43 [-0.88, 0.02]
**Subtotal (95% CI)**			**306**			**288**	**24.4%**	**-0.61 [-1.00, -0.23]**
Heterogeneity: Tau² = 0.09; Chi² = 8.42, df = 3 (P = 0.04); I² = 64%								
Test for overall effect: Z = 3.11 (P = 0.002)								
**4.1.4 Diarrhoea on day 4**								
Abolfazl 2018	11.1	32.3	86	7.1	43.4	87	0.1%	4.00 [-7.39, 15.39]
Ener 2013	2.18	1.6	113	2.64	2.3	96	6.3%	-0.46 [-1.01, 0.09]
Vandenplas 2011	2	1.48	57	2	1.48	54	6.3%	0.00 [-0.55, 0.55]
**Subtotal (95% CI)**			**256**			**237**	**12.6%**	**-0.23 [-0.61, 0.16]**
Heterogeneity: Tau² = 0.00; Chi² = 1.88, df = 2 (P = 0.39); I² = 0%								
Test for overall effect: Z = 1.15 (P = 0.25)								
**4.1.5 Diarrhoea on day 5**								
Ener 2013	1.91	1	113	1.76	1.61	96	6.9%	0.15 [-0.22, 0.52]
**Subtotal (95% CI)**			**113**			**96**	**6.9%**	**0.15 [-0.22, 0.52]**
Heterogeneity: Not applicable								
Test for overall effect: Z = 0.79 (P = 0.43)								
**Total (95% CI)**			**1569**			**1386**	**100.0%**	**-0.43 [-0.78, -0.09]**
Heterogeneity: Tau² = 0.42; Chi² = 116.86, df = 19 (P < 0.00001); I² = 84%								
Test for overall effect: Z = 2.45 (P = 0.01)								
Test for subgroup differences: Chi² = 8.24, df = 4 (P = 0.08), I² = 51.4%								

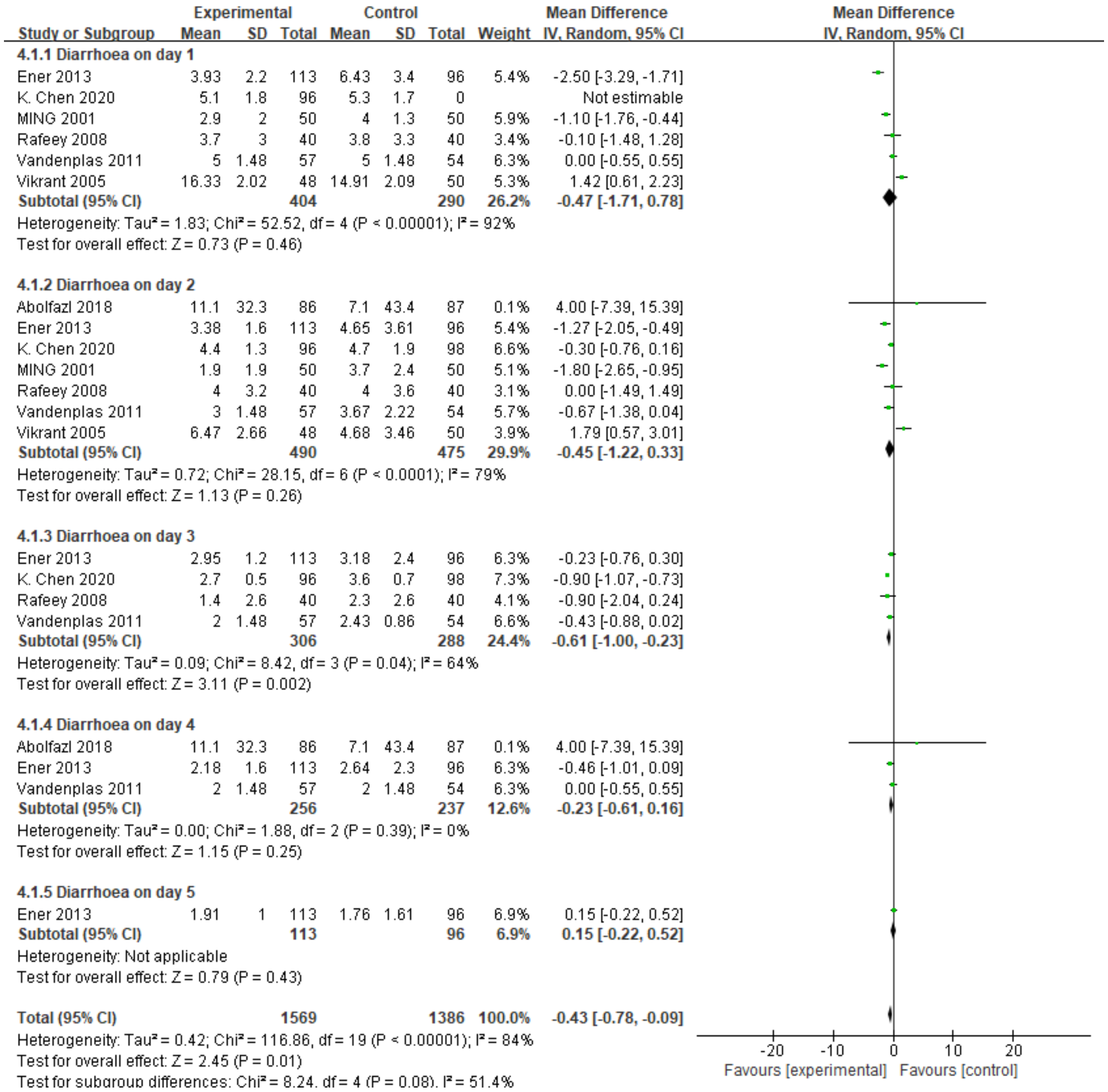

Figure S11. *Lactobacillus acidophilus* vs. control. Frequency of diarrhoea

References

1. Boulloche, J.; Mouterde, O.; Mallet, E. Management of acute diarrhea in infants and toddlers. Controlled study of the antidiarrheal efficacy of killed Lactobacillus acidophilus (LB strain) versus a placebo and a reference agent (loperamide). *Ann Pediatr (Paris).* **1994,** 41, 457–63. [CrossRef]
2. Han, H. J.; et al. Efficacy of Lactobacillus Acidophilus in Treatment of Acute Diarrhea in Children. *Korean journal of pediatric gastroenterology and nutrition.* **2004**, 7, 24-30.
3. Río, M.E.; Zago, L.B.; Garcia, H.; Winter, L. Influencia del estado nutricional sobre la efectividad de un suplemento dietario de bacterias lácticas. Prevencion y cura de diarreas infantiles [Influence of nutritional status on the effectiveness of a dietary supplement of live lactobacillus to prevent and cure diarrhoea in children]. *Arch Latinoam Nutr*. **2004**, 54, 287-92. [PubMed]
4. Rerksuppaphol, S.; Rerksuppaphol, L. Lactobacillus acidophilus and Bifidobacterium bifidum stored at ambient temperature are effective in the treatment of acute diarrhoea. *Ann Trop Paediatr*. **2010,** 30, 299-304. [CrossRef][PubMed]
5. Grandy, G.; Medina, M.; Soria, R.; Terán, C.G.; Araya, M. Probiotics in the treatment of acute rotavirus diarrhoea. A randomized, double-blind, controlled trial using two different probiotic preparations in Bolivian children. *BMC Infect Dis*. **2010**, 25, 10:253. [CrossRef][PubMed]
6. Pinto, J.M.; Petrova, A. Lactobacillus acidophilus Mixture in Treatment of Children Hospitalized With Acute Diarrhea. *Clin Pediatr (Phila)*. **2016**, 55(13), 1202-1209. [CrossRef][PubMed]
7. Kolader, M.E.; Vinh, H. Ngoc Tuyet, P.T.; Thompson, C.; Wolbers, M.; Merson, L.; Campbell, J.I.; Ngoc Dung, T.T.; Manh Tuan, H. Vinh Chau, N.V.; Farrar, J.; van Doorn, H.R.; Baker, S. An oral preparation of Lactobacillus acidophilus for the treatment of uncomplicated acute watery diarrhoea in Vietnamese children: study protocol for a multicentre, randomised, placebo controlled trial. *Trials.* **2013,** 28, 14:27. [CrossRef][PubMed]
8. Veereman-Wauters, G.; Staelens, S.; Wijffels, J. Pediatric Intervention Study With a Probiotic Mixture (Bacilac Forte) in Acute Gastroenteritis. *Journal of Pediatric Gastroenterology & Nutrition.* **2009**, 48, S114. [CrossRef]

Section and Topic	Item #	Checklist item	Location where item is reported
**TITLE**			
Title	1	dentify the report as a systematic review, meta-analysis, or both.	1
**ABSTRACT**			
Abstract	2	Provide a structured summary including, as applicable:<br>**Background:** main objectives<br>**Methods:** data sources; study eligibility criteria, participants, and interventions; study appraisal; and synthesis methods, such as meta-analysis.<br>**Results:** number of studies and participants identified; Authors may choose to summarize pairwise comparisons against a chosen intervention included in their analyses.<br>**Discussion/Conclusions:** limitations; conclusions and implications of findings.<br>**Other:** primary source of funding; systematic review registration number.	1
**INTRODUCTION**			
Rationale	3	Describe the rationale for the review in the context of existing knowledge.	2
Objectives	4	Provide an explicit statement of the objective(s) or question(s) the review addresses.	2
**METHODS**			
Eligibility criteria	5	Specify the inclusion and exclusion criteria for the review and how studies were grouped for the syntheses.	2
Information sources	6	Specify all databases, registers, websites, organisations, reference lists and other sources searched or consulted to identify studies. Specify the date when each source was last searched or consulted.	2
Search strategy	7	Present the full search strategies for all databases, registers and websites, including any filters and limits used.	2
Selection process	8	Specify the methods used to decide whether a study met the inclusion criteria of the review, including how many reviewers screened each record	2

Section and Topic	Item #	Checklist item	Location where item is reported
		and each report retrieved, whether they worked independently, and if applicable, details of automation tools used in the process.	
Data collection process	9	Specify the methods used to collect data from reports, including how many reviewers collected data from each report, whether they worked independently, any processes for obtaining or confirming data from study investigators, and if applicable, details of automation tools used in the process.	2,3
Data items	10a	List and define all outcomes for which data were sought. Specify whether all results that were compatible with each outcome domain in each study were sought (e.g. for all measures, time points, analyses), and if not, the methods used to decide which results to collect.	3
	10b	List and define all other variables for which data were sought (e.g. participant and intervention characteristics, funding sources). Describe any assumptions made about any missing or unclear information.	3
Study risk of bias assessment	11	Specify the methods used to assess risk of bias in the included studies, including details of the tool(s) used, how many reviewers assessed each study and whether they worked independently, and if applicable, details of automation tools used in the process.	3
Effect measures	12	Specify for each outcome the effect measure(s) (e.g. risk ratio, mean difference) used in the synthesis or presentation of results.	3
Synthesis methods	13a	Describe the processes used to decide which studies were eligible for each synthesis (e.g. tabulating the study intervention characteristics and comparing against the planned groups for each synthesis (item #5)).	3
	13b	Describe any methods required to prepare the data for presentation or synthesis, such as handling of missing summary statistics, or data conversions.	3
	13c	Describe any methods used to tabulate or visually display results of individual studies and syntheses.	3
	13d	Describe any methods used to synthesize results and provide a rationale for the choice(s). If meta-analysis was performed, describe the model(s), method(s) to identify the presence and extent of statistical heterogeneity, and software package(s) used.	3
	13e	Describe any methods used to explore possible causes of heterogeneity among study results (e.g. subgroup analysis, meta-regression).	3,4
	13f	Describe any sensitivity analyses conducted to assess robustness of the synthesized results.	3

Section and Topic	Item #	Checklist item	Location where item is reported
Reporting bias assessment	14	Describe any methods used to assess risk of bias due to missing results in a synthesis (arising from reporting biases).	3
Certainty assessment	15	Describe any methods used to assess certainty (or confidence) in the body of evidence for an outcome.	3
**RESULTS**			
Study selection	16a	Describe the results of the search and selection process, from the number of records identified in the search to the number of studies included in the review, ideally using a flow diagram.	4
	16b	Cite studies that might appear to meet the inclusion criteria, but which were excluded, and explain why they were excluded.	5
Study characteristics	17	Cite each included study and present its characteristics.	5
Risk of bias in studies	18	Present assessments of risk of bias for each included study.	5
Results of individual studies	19	For all outcomes, present, for each study: (a) summary statistics for each group (where appropriate) and (b) an effect estimate and its precision (e.g. confidence/credible interval), ideally using structured tables or plots.	5,6
Results of syntheses	20a	For each synthesis, briefly summarise the characteristics and risk of bias among contributing studies.	5,6
	20b	Present results of all statistical syntheses conducted. If meta-analysis was done, present for each the summary estimate and its precision (e.g. confidence/credible interval) and measures of statistical heterogeneity. If comparing groups, describe the direction of the effect.	7,8,9
	20c	Present results of all investigations of possible causes of heterogeneity among study results.	7,8,9
	20d	Present results of all sensitivity analyses conducted to assess the robustness of the synthesized results.	9

Section and Topic	Item #	Checklist item	Location where item is reported
Reporting biases	21	Present assessments of risk of bias due to missing results (arising from reporting biases) for each synthesis assessed.	5
Certainty of evidence	22	Present assessments of certainty (or confidence) in the body of evidence for each outcome assessed.	7,8,9
**DISCUSSION**			
Discussion	23a	Provide a general interpretation of the results in the context of other evidence.	9,10
	23b	Discuss any limitations of the evidence included in the review.	11,12
	23c	Discuss any limitations of the review processes used.	11,12
	23d	Discuss implications of the results for practice, policy, and future research.	12
**OTHER INFORMATION**			
Registration and protocol	24a	Provide registration information for the review, including register name and registration number, or state that the review was not registered.	2
	24b	Indicate where the review protocol can be accessed, or state that a protocol was not prepared.	not prepared
	24c	Describe and explain any amendments to information provided at registration or in the protocol.	No
Support	25	Describe sources of financial or non-financial support for the review, and the role of the funders or sponsors in the review.	13
Competing interests	26	Declare any competing interests of review authors.	12
Availability of data, code and other materials	27	Report which of the following are publicly available and where they can be found: template data collection forms; data extracted from included studies; data used for all analyses; analytic code; any other materials used in the review.	12,13

From: Page MJ, McKenzie JE, Bossuyt PM, Boutron I, Hoffmann TC, Mulrow CD, et al. The PRISMA 2020 statement: an updated guideline for reporting systematic reviews. BMJ 2021;372:n71. doi: 10.1136/bmj.n71

For more information, visit: http://www.prisma-statement.org/
